# Glucose control in intensive care: usability, efficacy and safety of Space GlucoseControl in two medical European intensive care units

**DOI:** 10.1186/1472-6823-14-62

**Published:** 2014-07-29

**Authors:** Karin Amrein, Norman Kachel, Heike Fries, Roman Hovorka, Thomas R Pieber, Johannes Plank, Urs Wenger, Barbara Lienhardt, Marco Maggiorini

**Affiliations:** 1 Medical University of Graz, Austria, Department of Internal Medicine, Division of Endocrinology and Metabolism, Auenbruggerplatz 15, 8036 Graz, Austria; 2 BBraun, Melsungen, Germany; 3 Institute of Metabolic Science, University of Cambridge, Cambridge, UK; 4 Joanneum Research Forschungsgesellschaft mbH, Graz, Austria; 5 Medical University of Zurich, Department of Internal Medicine, Medical Intensive Care Unit, Zurich, Switzerland

**Keywords:** Tight glycemic control, Critical illness, Critically ill patients, Protocol, Computer-assisted glycemic control, Insulin infusion protocol, Glucose control in intensive care

## Abstract

**Background:**

The Space GlucoseControl system (SGC) is a nurse-driven, computer-assisted device for glycemic control combining infusion pumps with the enhanced Model Predictive Control algorithm (B. Braun, Melsungen, Germany). We aimed to investigate the performance of the SGC in medical critically ill patients.

**Methods:**

Two open clinical investigations in tertiary centers in Graz, Austria and Zurich, Switzerland were performed. Efficacy was assessed by percentage of time within the target range (4.4-8.3 mmol/L; primary end point), mean blood glucose, and sampling interval. Safety was assessed by the number of hypoglycemic episodes (≤2.2 mmol/L) and the percentage of time spent below this cutoff level. Usability was analyzed with a standardized questionnaire given to involved nursing staff after the trial.

**Results:**

Forty medical critically ill patients (age, 62 ± 15 years; body mass index, 30.0 ± 8.9 kg/m^2^; APACHE II score, 24.8 ± 5.4; 27 males; 8 with diabetes) were included for a period of 6.5 ± 3.7 days (n = 20 in each center). The primary endpoint (time in target range 4.4 to 8.3 mmol/l) was reached in 88.3% ± 9.3 of the time and mean arterial blood glucose was 6.7 ± 0.4 mmol/l. The sampling interval was 2.2 ± 0.4 hours. The mean daily insulin dose was 87.2 ± 64.6 IU. The adherence to the given insulin dose advice was high (98.2%). While the percentage of time spent in a moderately hypoglycemic range (2.2 to 3.3 mmol/L) was low (0.07 ± 0.26% of the time), one severe hypoglycemic episode (<2.2 mmol/L) occurred (2.5% of patients or 0.03% of glucose readings).

**Conclusions:**

SGC is a safe and efficient method to control blood glucose in critically ill patients as assessed in two European medical intensive care units.

## Background

Since more than a decade, glucose control (GC) has been an important, yet laborious treatment goal in intensive care. After the first reactions that followed the landmark Leuven trial in 2001 [1] and led to the widespread attempt to implement tight glycemic control (TGC, target 4.4-6.1 mmol/L) in intensive care units (ICUs) worldwide, enthusiasm rapidly tapered when it became clear that safe and efficient GC requires experience, time and appropriate training besides being time-consuming and hard to achieve, even in the controlled setting of clinical trials.

Poor glycemic control - represented by hyperglycemia, hypoglycemia and high variability - is strongly and consistently associated with poor clinical outcomes [2-5], although the effect seems to be attenuated in patients with diabetes [5,6]. In one of the largest observational databases reported to date, Badawi and colleagues demonstrated in almost 200,000 critically ill patients that mortality was lowest in a blood glucose range between 4.4-6.1 mmol/L and progressively increased with severity and duration of hyperglycemia, hypoglycemia and with higher variability [7]. However, due to the high risk of hypoglycemia, this target range is no longer universally recommended and guidelines currently advocate less strict glycemic control in the setting of critical illness and perioperative care [8-13]. It seems reasonable to at least prevent severe hyperglycemia (>10 mmol/L) [14] that may lead to glucosuria and fluid dysbalance.

High-quality GC can best be achieved with a protocol combining continuous intravenous insulin with frequent blood glucose measurements. In the last decade, most ICUs have implemented nurse-based protocols, but their use is often restricted by their complexity and the inability to accurately account for changes in nutrition, an established risk factor for the occurrence of hypoglycemic episodes [15]. Computer-assisted GC may be able to overcome some of the difficulties encountered in daily routine and appears to be superior to standard care [16-21]. In the hope of improving the quality of GC and reducing workload, several algorithms have been developed. The enhanced Model Predictive Control (eMPC) algorithm has been studied in several clinical trials where it was found to be efficient and safe [22-27]. It is now implemented in the CE-certified Space GlucoseControl (SGC; B. Braun, Melsungen, Germany) which was the first device on the market available for routine use. We therefore aimed to test this nurse-driven device in two medical intensive care units (ICUs). The primary objective was to investigate the efficacy of the system defined as time in target range using a broader target range of 4.4-8.3 mmol/L for glucose control in medical ICU patients.

## Methods

The study was performed as a non-controlled clinical investigation in 40 medical critically ill patients at two tertiary academic centers (Medical University of Graz, Austria and the Medical University of Zurich, Switzerland). The trial was registered at the Clinical Trials Database (ClinicalTrials.gov Identifier: NCT01164423 and NCT01164449). Selected data of the trial in Graz have been published previously [28].

### Informed consent procedure

Both institutional ethics committees (EC) approved the trial before commencement, but the informed consent procedure differed: at the Medical University of Graz, the EC provided surrogate informed consent for study inclusion. Patients who regained consciousness provided written informed consent afterwards. At the Medical University of Zurich, the relatives of the patient and one physician uninvolved with routine treatment of the patient had to consent before any study-related activities. The trial was conducted according to Declaration of Helsinki and ISO 14155.

### Study population

Adult medical ICU patients assumed to stay ≥ 72 hours at the ICU were screened for inclusion (>6.1 mmol/l or already on insulin therapy) and exclusion criteria (insulin allergy, presence of ketoacidosis, moribund patients likely to die within 24 hours). All 40 screened patients were enrolled and analyzed.

### Target range and relevant variables for model prediction

The chosen target range for this trial was 4.4 - 8.3 mmol/l. Variables included in the individualized model prediction of insulin resistance are body weight, glucose concentration, administered insulin and carbohydrates (via enteral and parenteral nutrition).

### Definition of hypoglycemia

Severe hypoglycemia was defined as < 2.2 mmol/l and moderate hypoglycemia as < 3.3 mmol/l.

### SGC system and eMPC algorithm: training and maintenance

The B. Braun Space GlucoseControl system (SGC) was run at the bedside by the ICU nurses. SGC consists of three infusion pumps, two for enteral and parenteral nutrition (B. Braun Infusomat® Space) and one for insulin (B. Braun Perfusor® Space). The pumps are interconnected via the Space Station that allows data communication between the pumps and the central user interface, a touchscreen (SpaceControl) attached to the insulin pump. The eMPC algorithm is implemented in the SGC Module attached to and controlled by SpaceControl. The handling of the eMPC algorithm has been described in detail previously [25,29]. In brief, SGC gives an alarm that the suggested time to the next sample has elapsed. A nurse then measures and enters a current blood glucose value in order to receive an advice regarding insulin dose and time for the next glucose measurement (between 30 and 240 minutes). The advised insulin dose rate has to be confirmed and is then set automatically at the pump. Changes in enteral and parenteral nutrition are directly communicated to the eMPC by the respective pumps that are part of SGC. Changes in nutrition automatically lead to an adopted insulin dose rate proposal. The system also stores all data on therapy, displays these data and trends all relevant information on the user interface. In the last decade, various institutions across Europe have actively participated in several trials using the eMPC algorithm in a laptop computer version and a prototype version of the SGC system [22-27,30,31]. Based on the results of these studies in different environments, changes were implemented in the algorithm.

The pumps used in SGC are the same as in routine use at the Medical University Graz (B. Braun Infusomat® and Perfusor® Space), while the Medical University Zurich currently uses other pumps. Therefore, training at the latter site was more extensive. In Graz, all actively participating nurses attended a structured one-on-one training on virtual patients before enrolment of the first patient o familiarize with the handling of the complete SGC system. In Zurich, during daytime the device was handled by a dedicated study nurse who also briefly trained routine nursing staff for SGC use during other times.

All trial related activities were carried out until referral to the general ward, loss of the arterial line, end of iv insulin need or after 14 treatment days. After termination of the study, all patients were followed up for one week. Glucose measurements were performed using an arterial line available for routine monitoring purposes. Glucose measurements were performed with a certified device for glucose measurement in the ICU (Graz: Accu-Check Inform, Roche Diagnostics GmbH, Mannheim, Germany; Zurich: Accu-Check Aviva®, Roche Diagnostics GmbH, Mannheim, Germany).

For intravenous insulin infusion, insulin aspart (Novorapid, Novo Nordisk, Baegsvard, Denmark) was used in Graz and recombinant human insulin (Actrapid, Novo Nordisk, Baegsvard, Denmark) was used in Zurich. After study completion, we asked the ICU nursing staff to fill in a questionnaire on user acceptance of SGC.

### Statistical analysis

Statistical analysis was performed on an intention to treat basis. Blood glucose values were linearly interpolated. The percentage of time within the predefined glucose the target range (4.4-8.3 mmol/l) was defined as primary endpoint for the assessment of glucose control. Data are reported as mean ± SD if not otherwise indicated. Data analysis was performed using SPSS® version 19.0.

## Results

### Study population and concomitant treatment

40 medical critically ill patients were included from February 2010 to August 2011 (Graz February to December 2010, Zurich August 2010 to August 2011). Up to four patients were treated with SGC systems simultaneously at each site. Baseline characteristics of the Zurich population (n = 20) are given in Table 1. Baseline characteristics of the Graz population have been reported previously [28] and are similar except for a higher rate of norepinephrine and parenteral nutrition. Because of substantial national differences in informed consent procedure for patients unable to give consent at the time of study inclusion, time from ICU admission to study inclusion was significantly different between the two sites (Graz 1.7 ± 1.5, Zurich 6.7 ± 5.9 days, P = 0.001). Before study start, blood glucose was controlled using the standard protocol.

**Table 1:** Demographic and clinical characteristis of the study population in Zurich (n = 20)

**Demographic characteristics**	
Male (n/%)	13 (65%)
Age (yrs)	60.2 ± 14.3
Body mass index (kg/m^2^)	29.0 ± 6.6
APACHE II	24.2 ± 4.5
**Clinical characteristics (n)**	
Admission diagnosis	Post cardiac arrest (4)
	Sepsis (3)
	Pulmonary (3)
	Cardiac (5)
	Neurologic (1)
	Other (4)
Mechanically ventilated	17 (85%)
Vasopressor therapy	Norepinephrine: 11 (55%) Dobutamine: 4 (20%)
Renal replacement therapy	4 (20%)
Steroid treatment	6 (30%)
Parenteral nutrition	6 (30%)
Enteral nutrition	18 (90%)
History of diabetes	4 (20%)
Patients on insulin before study start	18 (90%)
Hospital mortality	6 (30%)

Data are given as numbers (n) or means ± SD as appropriate. Baseline data of the study population in Graz have been reported previously [28]. There are no significant differences between both populations with the exception of a higher rate of norepinephrine use (90 vs. 55%, P < 0.01) and a higher rate of parenteral nutrition in Graz 85 vs. 30%, P < 0.01.

### Protocol adherence, glucose control and sampling frequency

**Figure 1:**
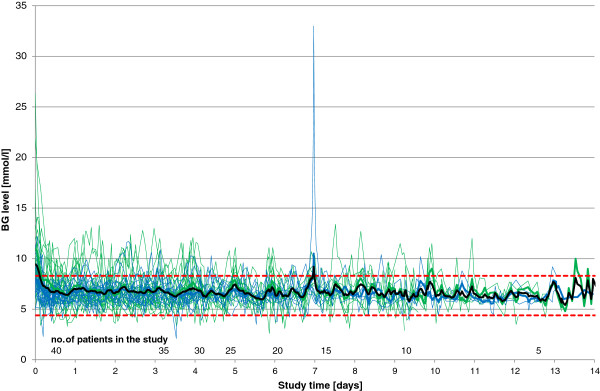
**Average (bold line) and individual (thin lines) glucose profiles.** Dashed lines mark the target range 4.4–8.3 mmol/L. Blue lines are Zurich data, green lines are Graz data. The peak on day 7 (33 mmol/L) was caused by treatment for acute hyperkalemia with a 20% dextrose bolus.

No major protocol violations occurred. Overall, the primary endpoint (time in target range 4.4 to 8.3 mmol/l) was reached in 83.4 ± 8.9% of the time in Graz and in 93.1 ± 7.1% in Zurich (p < 0.01). The mean glucose level was not significantly different between both sites (6.8 ± 0.4 vs. 6.6 ± 0.4 mmol/l). At study start, the mean glucose level was 9.4 ± 4.2 mmol/l (range: 4.8 to 26.3 mmol/l). The daily number of glucose sampling varied from 8 to 19 times (12 ± 2) and the mean sampling interval per day varied from 1.3 to 3.0 hours (2.2 ± 0.4). Detailed information regarding glucose readings and sampling intervals within each center is given in Table 2. Percentages of time per day within blood glucose level ranges with a focus on Zurich data are given in Table 3, the detailed Graz data have been reported previously [28]. Average and individual glucose profiles are displayed in Figure 1. Patients were included for 6.5 ± 3.7 days in the trial.

**Table 2:** Important details of glucose control and sampling interval during the study

	**Graz**	**Zurich**	**P**	**Total**
**Blood glucose level at study start [mmol/l]**	10.9 ± 5.3	8.0 ± 1.7	0.021	9.4 ± 4.2
**Time from ICU admission to study start [days]**	1.7 ± 1.5	6.7 ± 5.9	0.001	4.2 ± 4.9
**Time to reach target range [hours]**	5.3 ± 4.6	2.9 ± 2.3	0.157	4.7 ± 4.1
**Sampling interval [hours]**	2.0 ± 0.4	2.3 ± 0.4	0.013	2.2 ± 0.4
**Mean BG level [mmol/l]**	6.8 ± 0.4	6.6 ± 0.4	0.142	6.7 ± 0.4
**< 3.3 mmol/l**	0.03 ± 0.07	0.11 ± 0.37	0.297	0.07 ± 0.26
**3.3-4.3 mmol/l**	2.06 ± 1.83	0.47 ± 0.64	0.002	1.26 ± 1.57
**4.4-8.3 mmol/l (target range)**	**83.4 ± 8.9**	**93.1 ± 7.1**	**0.060**	**88.3 ± 9.3**
**> 8.3 mmol/l**	14.4 ± 16.7	7.6 ± 16.5	0.159	10.4 ± 8.5
**Total study time [days]**	7.0 ± 3.6	6.0 ± 3.8	0.389	6.5 ± 3.7

Data are given as mean ± SD. Numbers for blood glucose ranges refer to percentage of time within the given range, boldface numbers give the results for the target range.

**Table 3:** Glucose control for individual study days with a focus on Zurich data

**Study day**	**n**	**Percentage of time [%]**			
		**<3.3 mmol/l**	**3.3-4.3 mmol/l**	**4.4-8.3 mmol/l**	**>8.3 mmol/l**
				**(Target range)**	
1	20	0.33 ± 1.50	0.9 ± 3.0	91.6 ± 10.6	7.2 ± 10.5
2	18	0	0	94.6 ± 8.0	5.4 ± 8.0
3	18	0	0.1 ± 0.5	92.3 ± 16.5	7.6 ± 16.5
4	15	0.5 ± 1.8	0.8 ± 2.2	91.4 ± 12.5	7.3 ± 11.8
5 and later	13	0	1.2 ± 2.8	94.5 ± 5.7	4.3 ± 5.3
**Total both sites**	**40**	**0.07 ± 0.26**	**1.3 ± 1.6**	**88.3 ± 9.3**	**10.4 ± 8.5**
**Total Graz**	**20**	**0.03 ± 0.07**	**2.1 ± 1.8**	**83.4 ± 8.9**	**14.5 ± 8.3**
**Total Zurich**	**20**	**0.11 ± 0.37**	**0.5 ± 0.6**	**93.1 ± 7.1**	**6.3 ± 6.6**

Data are given as means ± SD. Detailed data of the study population in Graz have been reported previously [28], boldface numbers give the results for the target range.

### Safety

6 of 40 patients (15%) experienced hypoglycemia (<3.3 mmol/l), this corresponded to 0.2% (6 out of 3044) of all measurements. The percentage of time within 2.2 and 3.3 mmol/l was low (0.07 ± 0.26%). One severe hypoglycemic episode (2.1 mmol/l) occurred during the trial, corresponding to 0.03% of all measurements or 2.5% of.all patients. In the previously stable patient, the blood glucose was at 6.5 mmol/l. SGC advised to continue the insulin rate of 6.4 U/h and to measure blood glucose again after 4 hours. After 90 minutes, the nurse measured blood glucose which was 4.5 mmol/l, but did not enter it into the SGC system. At the proposed time, the blood glucose of the patient was 2.1 mmol/l. The affected patient had been mostly within target range for 3 days before this incident, but after the insulin requirement was remarkably lower. No relevant nutrition changes were undertaken during this period.

### Insulin and nutrition

Daily average insulin requirement was 87.2 ± 64.6 IU (range: 18 to 360 IU). Virtually all patients received enteral nutrition via continuous infusion (38 of 40; 95%) and the majority also received parenteral feeding (23 of 40; 57.5%).

### User interventions and usability

The adherence to the given insulin dose advice was high (98.2%). Out of 3285 eMPC advices, the user overruled in 59 (1.8%) times: in 54 cases downwards (1.64%) and 5 times upwards (0.15%). There was no significant difference between the study sites. The magnitude of deviations ranged from 0.1 to 18.0 IU insulin/hour and the major reasons for overruling included small or large changes in the advised insulin dose (e.g. ICU nurses set a rate of or 0.0 IU instead of 0.6, or 5 IU insulin/hour instead of proposed 15) and simply feeling uneasy with the proposed insulin rate. The sampling time was excellently followed.

At the end of the study, the nursing staff was asked to complete a questionnaire regarding the usability of SGC. 51 of 100 involved nurses returned the questionnaire (18/64 or 28% in Zurich, 31/46 or 67% in Graz).

There were significant differences in the subjective judgement between the two centers, as the nursing staff in Graz answered all of the 9 questions significantly more favorably than in Zurich (Table 4).

**Table 4:** Questionnaire results

**Questionnaire, answers in favour of Space TGC**	**Total (N = 51)**		**Graz (N = 33)**		**Zürich (N = 18)**		**P**
	**Number**	**Rate**	**Number**	**Rate**	**Number**	**Rate**	
1: Performance (“good” or “excellent”)	35	68.6%	27	81.8%	8	44.4%	0.011
2: Reduction of workload	12	23.5%	11	33.3%	1	5.6%	0.037
3: Efficacy	33	64.7%	31	93.9%	2	11.1%	< 0.001
4: User friendlyness	34	66.7%	30	90.9%	4	22.2%	< 0.001
5: Problems in use	29	56.9%	24	72.7%	5	27.8%	0.003
6: Confidence	35	68.6%	29	87.9%	6	33.3%	< 0.001
7: Prevention of mistakes	33	64.7%	28	84.8%	5	27.8%	< 0.001
8: Routine use	25	49.0%	23	69.7%	2	11.1%	< 0.001
9: Reliability (“complete trust”)	23	45.1%	21	63.6%	2	11.1%	< 0.001

Nursing staff at both sites was asked to complete a questionnaire after recruitment had finished. These were the original formulations:

1. “How well was the patient’s blood glucose stabilised in the target range from 4.4 and 8.3 mmol/l?”

2. “Was your workload using Space TGC System reduced or increased?”

3. “Do you think that control of the patient’s blood glucose was maintained more effectively by the use of the Space TGC system compared with normal practice?”

4. “Is the Space TGC system user friendly?”

5. “Were there any problems with the use of the Space TGC system?”

6. “Did you feel confident using the Space TGC system?”

7. “Do you think that using the Space TGC system can help to avoid making mistakes when controlling the blood glucose?”

8. “Do you think that the Space TGC system may be a worthwhile tool for routine use in the ICU?”

9. “Did you trust the Space TGC system would maintain the blood glucose at a safe level?”

## Discussion

The debate about ideal glycemic control in intensive care continues. In the last years, large intervention studies including the NICE-SUGAR trial unexpectedly failed to replicate the initial and very promising finding of the first Leuven study showing a significant survival benefit for TGC in surgical ICU patients [1,32,33]. Substantial differences between these studies were present in study design, execution of glucose control and treated population. Likely one of the main reasons why clinical trials and meta-analyses showed negative results for TGC was the high incidence of hypoglycemia induced by intensive insulin therapy. Probably it was impossible to prevent this because no reliable tool facilitating GC was available. The used (paper-based) protocol was often not extensively tested before implementation, and adherence to it was not reported or registered, a fact that may be especially problematic in a multi-center approach where different sites often display substantial differences in standards of critical care including important aspects in nutrition and glycemic control. Despite all efforts, a large gap is still evident between what is achievable and what is desirable in glucose control – (near) normoglycemia besides minimal variability [34].

SGC with the implemented eMPC algorithm is a well-validated, CE-certified nurse-driven tool to assist glucose control in critical care. As demonstrated in our two centers, it was possible to achieve excellent adherence and glycemic control with low variability. The target range of 4.4 to 8.3 mmol/l could be achieved in 83% (Graz) and 93% (Zurich) of the time. Despite excellent efficacy, usability and performance parameters were assessed very differently by the nursing staff at the two study sites. In Zurich, glucose control was outstanding and significantly better than in Graz, yet the involved ICU staff turned out to be unsatisfied. We hypothesize that this difference was caused by 1) the intensive hands-on simulated training performed only in Graz and 2) the long lasting routine use of BBraun pumps at the same site. Although the training was a time-consuming and laborious process, it nevertheless seems to be necessary when implementing such a new tool in order to assure long-term functionality and operator satisfaction within the ICU team. The fact that infusion pumps from another manufacturer are in use in Zurich meant that ICU staff needed to become familiar with even more new equipment and had to handle two different types of pumps at the same time. This is particularly evident in the response to question 8 regarding potential routine use which was viewed favorably by 70% of nurses in Graz, but only 11% in Zurich.

Workload was perceived to be increased by the majority of the users which is explainable by the relatively frequent sampling and high documentation effort compared to current routine care at both sites.

With regard to safety, one patient experienced severe hypoglycemia (2.5% of patients or 0.03% of glucose readings) which seems an acceptable rate in this population. This incident was primarily attributable to a long (4-hour) measurement interval that was suggested by the device. However, although the responsible nurse felt uneasy and measured a dropping blood glucose level of 4.5 mmol/l, it was not entered in the SGC and insulin infusion continued with the same rate. With more extensive training of the involved staff, this hypoglycemic episode may have been prevented as the eMPC almost certainly would have advised to stop insulin infusion in view of a rapidly falling blood glucose concentration. The continued use of insulin at a glucose level below 5.6 mmol/l has recently been identified as one of the most common causes for hypoglycemia in an analysis of > 55,000 glucose readings in 1,657 patients [17]. This incident also demonstrates that the possibility of user overruling may be crucial for optimal SGC use and the device’s suggestions should only be followed in view of the clinical context.

Obviously, our study is limited by its small sample size and open design. However, the primary aim was to in detail evaluate the complete SGC system in a medical population of critically ill patients at two different sites. As demonstrated by the individual profiles of the participants, glucose control could be established and maintained over the whole study period in both centers. Another limitation is the use of bedside glucometers as they were originally developed for glucose measurement in another setting and are not accurate enough for glycemic control in critically ill patients, especially when anemia is present [35,36]. Morever, the mean sampling interval of > 2 hours is a potential limitation as in comparison with continuous glucose monitoring, hypo- and hyperglycemic excursions of blood glucose may have been missed, as also described in the literature [37]. Certainly, our observations need to be confirmed in larger populations with a focus on presence or absence of diabetes and also in a setting with CGM.

Under clinical trial conditions, subcutaneous continuous glucose monitoring (CGM) has been found to be sufficiently accurate when compared to arterial sampling [38]. In a small pilot study, Kopecky and colleagues showed that combining the eMPC algorithm with CGM is a feasible method for glycemic control in cardiac surgery patients [39]. Okabayashi et al. could demonstrate a reduced risk for surgical site infections and excellent perioperative tight glycemic control without hypoglycemic episodes using a closed loop glycemic control system with CGM in patients requiring hepato-biliary-pancreatic surgery [40].

These studies and ours are important steps on the way to the artificial pancreas at the bedside of the ICU patient - a real closed loop system that would ideally combine reliable continuous glucose sampling with a semi-automated method to improve glycemic control and reduce workload [41]. Major efforts are also being undertaken to internationally standardize important aspects of glycemic control, as evident in the 2013 Consensus recommendations jointly written by the leading experts in the field [42]. These developments will certainly greatly contribute in the implementation of safe, efficient and not overly elaborate glucose control in critical care.

## Conclusions

SGC is a reliable and efficient computerized method for glycemic control in the ICU. We suggest that SGC may be a useful tool to aid in routine glycemic control and reduce disparities in standards of care when performing future multicenter clinical trials. Such trials are without doubt necessary to address important details in the blood glucose management of critically ill patients, such as the use of individualized target ranges for different patient populations, especially for diabetics versus non-diabetics [43]. However, our experience indicates that extensive training before implementation of such a tool is crucial in order to ascertain the compliance of operators and to minimize hypoglycemic events.

## Competing interests

Karin Amrein and Marco Maggiorini received lecture fees, Johannes Plank received consultancy fees and Roman Hovorka received both lecture and consultancy fees from BBraun. Heike Fries and Norman Kachel are BBraun employees.

All other authors declare that they have no competing interests.

## Authors’ contributions

KA, JP and MM designed the study, drafted the manuscript and carried out the clinical part of the study including data collection. UW and BL recruited patients. NK participated in study design, drafting the manuscript and performed the statistical analysis. HF coordinated the study activities and statistical analysis. TP and RH revised the manuscript critically for important intellectual content. All authors read and approved the final manuscript.

## Pre-publication history

The pre-publication history for this paper can be accessed here:

http://www.biomedcentral.com/1472-6823/14/62/prepub
